# The Race for Hydroxamate-Based Zirconium-89 Chelators

**DOI:** 10.3390/cancers13174466

**Published:** 2021-09-04

**Authors:** Irene V. J. Feiner, Marie Brandt, Joseph Cowell, Tori Demuth, Daniëlle Vugts, Gilles Gasser, Thomas L. Mindt

**Affiliations:** 1 Ludwig Boltzmann Institute Applied Diagnostics, General Hospital of Vienna, 1090 Vienna, Austria; Irene.Feiner@lbiad.lbg.ac.at (I.V.J.F.); Marie.Brandt@lbiad.lbg.ac.at (M.B.); 2 Division of Nuclear Medicine, Department of Biomedical Imaging and Imaging Guided Therapy, Medical University of Vienna, 1090 Vienna, Austria; 3 Institute of Chemistry for Life and Health Sciences, Laboratory for Inorganic Chemical Biology, Chimie ParisTech, PSL University, CNRS, 75005 Paris, France; joseph.cowell@chimieparistech.psl.eu (J.C.); gilles.gasser@chimie-paristech.fr (G.G.); 4 TU Wien, Institut für Angewandte Synthesechemie, Getreidemarkt 9, 1060 Wien, Austria; tori.demuth@tuwien.ac.at; 5 TU Wien, Center for Labeling and Isotope Production, Stadionallee 2, 1020 Wien, Austria; 6 Department of Radiology and Nuclear Medicine, Amsterdam UMC, VU University Amsterdam, 1081 HV Amsterdam, The Netherlands; d.vugts@amsterdamumc.nl; 7 Department of Chemistry, Institute of Inorganic Chemistry, University of Vienna, Währinger Straße 42, 1090 Vienna, Austria

**Keywords:** hydroxamate chelators, zirconium-89, immunoPET, desferrioxamine, DFO

## Abstract

**Simple Summary:**

Chelators are small molecules that can form a complex with a metal ion by coordinating electron rich atoms from the chelator to the electron-poor cation. Bifunctionalization of the chelator allows for the coupling of the chelator to a vector, such as a biomolecule. Using this approach, radiolabeling of biomolecules with metallic radionuclides can be performed, enabling nuclear imaging studies for diagnosis and radiotherapy of diseases. In the case of positron emission tomography (PET) of radiolabeled antibodies, this approach is called immunoPET. In this review we focus on chelators using hydroxamate groups to coordinate the radionuclide zirconium-89 ([^89^Zr]Zr^4+^, denoted as ^89^Zr in the following). The most common chelator used in this context is desferrioxamine (DFO). However, preclinical studies indicate that the ^89^Zr-DFO complex is not stable enough in vivo, in particular when combined with biomolecules with slow pharmacokinetics (e.g., antibodies). Subsequently, new chelators with improved properties have been developed, of which some show promising potential. The progress is summarized in this review.

**Abstract:**

Metallic radionuclides conjugated to biological vectors via an appropriate chelator are employed in nuclear medicine for the diagnosis (imaging) and radiotherapy of diseases. For the application of radiolabeled antibodies using positron emission tomography (immunoPET), zirconium-89 has gained increasing interest over the last decades as its physical properties (t_1/2_ = 78.4 h, 22.6% β^+^ decay) match well with the slow pharmacokinetics of antibodies (t_biol_. = days to weeks) allowing for late time point imaging. The most commonly used chelator for ^89^Zr in this context is desferrioxamine (DFO). However, it has been shown in preclinical studies that the hexadentate DFO ligand does not provide ^89^Zr-complexes of sufficient stability in vivo and unspecific uptake of the osteophilic radiometal in bones is observed. For clinical applications, this might be of concern not only because of an unnecessary dose to the patient but also an increased background signal. As a consequence, next generation chelators based on hydroxamate scaffolds for more stable coordination of ^89^Zr have been developed by different research groups. In this review, we describe the progress in this research field until end of 2020, including promising examples of new candidates of chelators currently in advanced stages for clinical translation that outrun the performance of the current gold standard DFO.

## 1. Introduction

**Figure 1:**
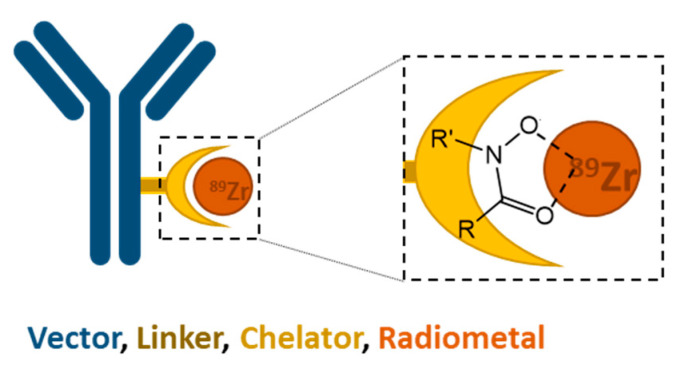
Schematic representation of a biological vector (antibody) conjugated via a linker to a hydroxamate chelator and radiolabeled with ^89^Zr. The enlargement shows the *O*,*O*-coordination between the electron rich oxygen atoms and the radiometal cation ^89^Zr forming a stable five-member-ring.

Nuclear imaging is a medical tool using radionuclides for diagnosis, to predict therapy outcome and follow disease progression. One distinct field of nuclear imaging, namely immunoPET, has become increasingly important over the last decade [1]. It combines the high target specificity of monoclonal antibodies (mAbs) with the high sensitivity of positron emission tomography (PET). The choice of an appropriate radioisotope for the labeling of mAbs relates to the following aspects. Besides the required positron decay for PET imaging, availability of the radionuclide and its physical half-life play a crucial role. Some of the most common PET isotopes, such as fluorine-18 (t_1/2_ = 109.7 min) and gallium-68 (t_1/2_ = 67.6 min), are not appropriate for immunoPET due to their short physical half-life which does not match the slow biodistribution of mAbs. Therefore, the use of long half-life radioisotopes, such as zirconium-89 (^89^Zr, t_1/2_ = 78.4 h) [2], iodine-124 (^124^I, t_1/2_ = 4.2 d) [3], copper-64 (^64^Cu, t_1/2_ = 12.7 h) [4], niobium-90 (^90^Nb, t_1/2_ = 14.6 h) [5] and manganese-52g (^52g^Mn, t_1/2_ = 5.6 d) [6], is more reasonable for radiolabeling of mAbs. However, while the availability of some of these radioisotopes is low, the production of ^89^Zr is rather straightforward via ^89^Y(p,n)^89^Zr reaction and be obtained commercially in sufficient purity [7,8]. That convenience makes ^89^Zr preferred and has led to a progressively larger set of data and publications. This rise of research related to ^89^Zr highlights the importance of finding an effective chelator for the stable complexation of the radiometal, since such is required for clinical applications of biomolecules radiolabeled with metallic radionuclides (Figure 1).

The use of chelators in radiolabeling approaches can be challenging as the vector usually undergoes bigger changes in its structure, compared to other labelling strategies, e.g., with a covalent ^124^I radiolabeling. However, when working with larger biomolecules (e.g., Abs) as in the case of immunoPET, the coupling of a chelator does usually not affect the bioactivity as long as the number of chelators per mAb molecule is moderate. A high number of conjugated chelators can lead to a reduced binding affinity of the Ab towards its respective antigen or induce aggregation of the protein. An advantage of the use of chelators is the commonly mild radiolabeling conditions (room temperature (RT), pH not deviating much from physiological pH, short reaction time) that can lead to a high radiochemical yield (RCY). Furthermore, the use of chelators and radiometals allow for the possibility of theranostic approaches by replacing a diagnostic radiometal with a therapeutic β^−^- or α-particle emitting metal [8].

The different chelators are typically grouped by the metal they can complex, their coordinating groups and their structure being macrocyclic or linear. Concerning the latter, macrocyclic chelators have usually a higher kinetic stability and furthermore a higher thermodynamic force to form a complex (macrocyclic effect) than acyclic chelators [9]. That mostly results from the preorganization of the macrocycle which leads to a smaller loss of entropy during the formation of the complex with a cation. Linear chelators have to undergo a much greater geometrical change. Yet in most cases, harsher conditions, such as heat and longer reaction time, are required for the complex formation of macrocyclic chelators. Such conditions are problematic in the context of sensitive biomolecules, such as Abs, where elevated temperatures will lead to denaturation of the protein.

It must be considered when developing a ligand for the coordination of a radionuclide that each metal ion has its unique properties, such as its aqueous coordination chemistry and redox chemistry [9]. In general, to evaluate a chelator, its ability to bind the metal ion in high yield is first tackled under close observation of the required conditions, such as pH, temperature and time. The determination of thermodynamic stability (K = [ML]/([M][L], with [LM] as the concentration of the ligand-metal-complex, [M] of the free metal, and [L] of the free ligand) provides a good possibility to compare different chelators. If the results of those preliminary studies are promising, in vitro assays (e.g., metal challenging experiments, blood serum stability) and finally in vivo tests (e.g., biodistribution experiments) must follow to ensure a highly stable and kinetically inert complex. For the latter, an appropriate biomolecule should be selected. A non-conjugated radiolabeled chelator is generally cleared too fast from the body to detect instabilities of the complex with slow release of the radiometal in vivo. Yet again, for ligands conjugated to biomolecules, the physical half-life of the radionuclide should correlate with the physiological half-life of the biomolecule (with exceptions [10]). A chelator for a nuclide with longer half-life (e.g., ^89^Zr) needs in vivo experiments over a longer time period (i.e., showing high complex stability over days in vivo) compared to a chelator that coordinates a radionuclide with shorter half-life (e.g., gallium-68 (^68^Ga)). In other words, high stability of the chelator-metal complex is important during the in vivo lifetime of the radiolabeled molecule.

## 2. ^89^Zr-Chelators Not Based on Hydroxamates

The majority of chelators used for ^89^Zr bear hydroxamate groups. The present review focusses therefore on this type of chelators for the stable complexation of ^89^Zr. However, this first section, appreciating the importance of other chelating systems, describes examples of structurally different chelators in nuclear medicine.

When ^89^Zr was first investigated for PET imaging, common universal metal chelators such as ethylenediaminetetraacetic acid (EDTA) and diethylenetriaminepentaacetic acid (DTPA) were used to explore the coordination chemistry and complex formation [11]. Their structures revealed the preferred coordination number is eight. For EDTA, being hexadentate, two water molecules are additionally involved in the complexation. DTPA, on the other hand, is able to saturate the coordination sphere of zirconium and achieves therefore higher thermodynamic stability. Like EDTA, the hydroxamate chelator desferrioxamine (DFO), is hexadentate, yet it shows superior chelating properties for zirconium than DTPA. The most probable explanation can be found in the hard and soft acids and bases (HSAB) concept. DTPA coordinates via three nitrogen and five oxygen atoms, whereas DFO offers six oxygen donors, which are favored by the hard oxophilic zirconium ion [11].

**Figure 2:**
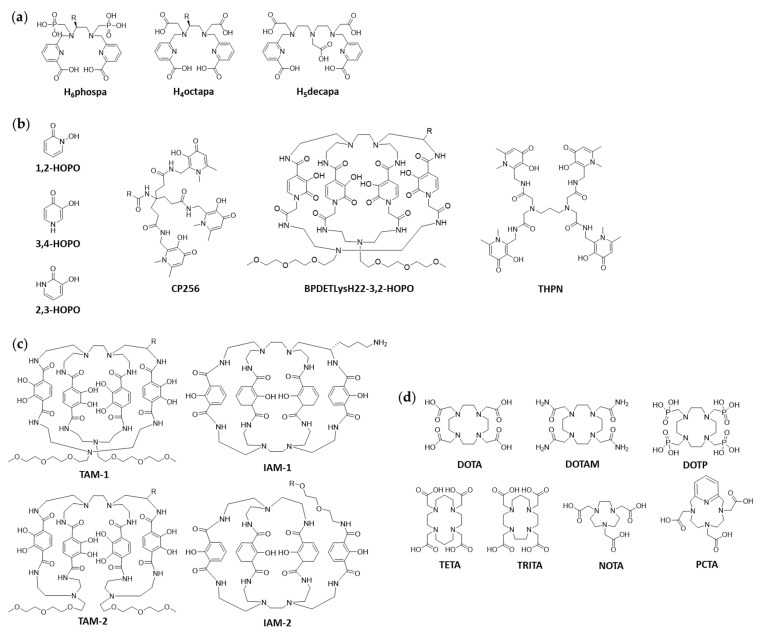
Exemplary structures for non-hydroxamate ^89^Zr-chelators. (**a**) Acyclic chelators of “pa” family. (**b**) HOPO-based chelators. (**c**) Terephthalamide- and hydroxyisophthalamide-based chelators. (**d**) Tetraazamacrocycle- and other polyazamacrocycle-based chelators. R represents location of bifunctionalization.

To name some more recent examples, the Orvig group has synthesized different acyclic chelators, the so-called pa” family (H_2_dedpa, H_4_octapa, H_2_azapa, and H_5_decapa, Figure 2a). Radiolabeling studies focused on indium-111 ([^111^In]In^3+^), but trials with lutetium-177 ([^177^Lu]Lu^3+^) and ^89^Zr were also conducted for some chelators. However, ^89^Zr showed only low labeling yields (highest yield achieved: 12% with H_6_phospa-trastuzumab, pH 7.4, 37 °C, 18 h) [12,13]. Hydroxypyridinones (HOPOs) represent another family of compounds with good chelating properties (Figure 2b). While the 1,2-HOPO chelators will be included in the main part of this review since these are hydroxamate analogs, the 2,3- and 3,4-HOPO ligands should be mentioned here due to their promising results. Ma et al. worked on a 3,4-HOPO chelator based on tripodal tris(hydroxypyridinone) for ^89^Zr, named CP256 (which is called YM103 when bifunctionalized with maleimide; Figure 2b). In vitro and in vivo experiments were compared to DFO analogs. Even though good radiolabeling results could be obtained (pH 6.5, >96%, 60 min, RT), in vivo experiments (C57B1/6j mice) showed significantly lower complex stability compared to DFO (accumulation in bones 3 d post injection (p.i.), 5% ID/g vs. 29% ID/g for ^89^Zr-DFO-trastuzumab and ^89^Zr-YM103-trastuzumab, respectively) [14]. In 2016, Tinianow et al. reported a macrocyclic chelator for ^89^Zr based on 2,3-HOPO (BPDETLysH22-3,2-HOPO; Figure 2b). Quantitative radiolabeling could be achieved quickly and under mild conditions (pH 7–7.5, 15 min, RT). Furthermore, in vivo studies (NIH Swiss mice bearing xenografts of SCOV3 cancer cells) were performed with antibody conjugates and compared to DFO-analogs. No significant difference between the 2,3-HOPO conjugates and the DFO controls with regard to tumor uptake was observed. However, liver uptake was higher over the full time of the experiment for the 2,3-HOPO conjugates, while following the same trends as their DFO analogs [15]. 

A closer investigation of the complexation chemistry of Zr^4+^ with the three HOPO isomers (1,2-HOPO, 2,3-HOPO and 3,4-HOPO) was published by Guérard et al. in 2017 [16]. Buchwalder et al. synthesized another 3,4-HOPO chelator. The ligand tetrapodal 3-hydroxy-4-pyridinone (THPN; Figure 2b) showed excellent labelling properties (quantitative yield, 10 min, RT, pH 7.5) and outperformed the ^89^Zr-DFO complex in a direct transchelation challenge (7 d, pH 5, 1.6% vs. 41.6% intact ^89^Zr-DFO and ^89^Zr-THPN complex, respectively). In vivo studies showed fast elimination of the complex without signs of demetalation. No bioconjugation of THPN has been performed yet, hence, no judgment can be given about the potential of this chelator [17]. Finally, Pandya et al. developed both di-macrocyclic terephthalamide ligands (TAM-1, TAM-2, Figure 2c) and di-macrocyclic hydroxyisophthalamide ligands (IAM-1, IAM-2; Figure 2c) for the complexation of ^89^Zr. Those octadentate ligands could be radiolabeled quantitatively and showed good in vitro stability. A comparison of ^89^Zr-TAM-1 and ^89^Zr-DFO yielded similar results in terms of accumulation of radioactivity in bones (72 h, 0.078% ID/g vs. 0.074% ID/g ^89^Zr-DFO and ^89^Zr-TAM-1, respectively). IAM-2 showed high complex stability in vitro and in vivo, yet to achieve good labelling yields, higher temperature and longer reaction time were required [18,19]. The same group also published novel results in 2017 with the chelator DOTA and derivatives thereof. Other than previously assumed, Pandya et al. demonstrates that tetraazamacrocycles (such as DOTA, DOTAM, DOTP; Figure 2d), form highly stable complexes with ^89^Zr, despite the fact that next to oxygen also nitrogen atoms are involved in the complex formation with the oxophilic zirconium ion. First attempts with ^89^Zr-oxalate were not successful but changing to either ^89^ZrCl_4_ or ^89^Zr-acetylacetonate brought success. A drawback to these small macrocyclic chelators is the requirement of elevated temperature (90 °C) for the radiolabeling [20]. In a recent paper the same group evaluated smaller macrocyclic chelators, such as NOTA, TRITA and PCTA (Figure 2d) in an effort to overcome the hurdle of elevated temperature. Indeed, NOTA and PCTA could be radiolabeled under mild conditions (37 °C) and the radiolabeled chelators (no bioconjugation data available) showed good in vitro and in vivo characteristics [21].

## 3. DFO and Its Bifunctional Variants

**Figure 3:**
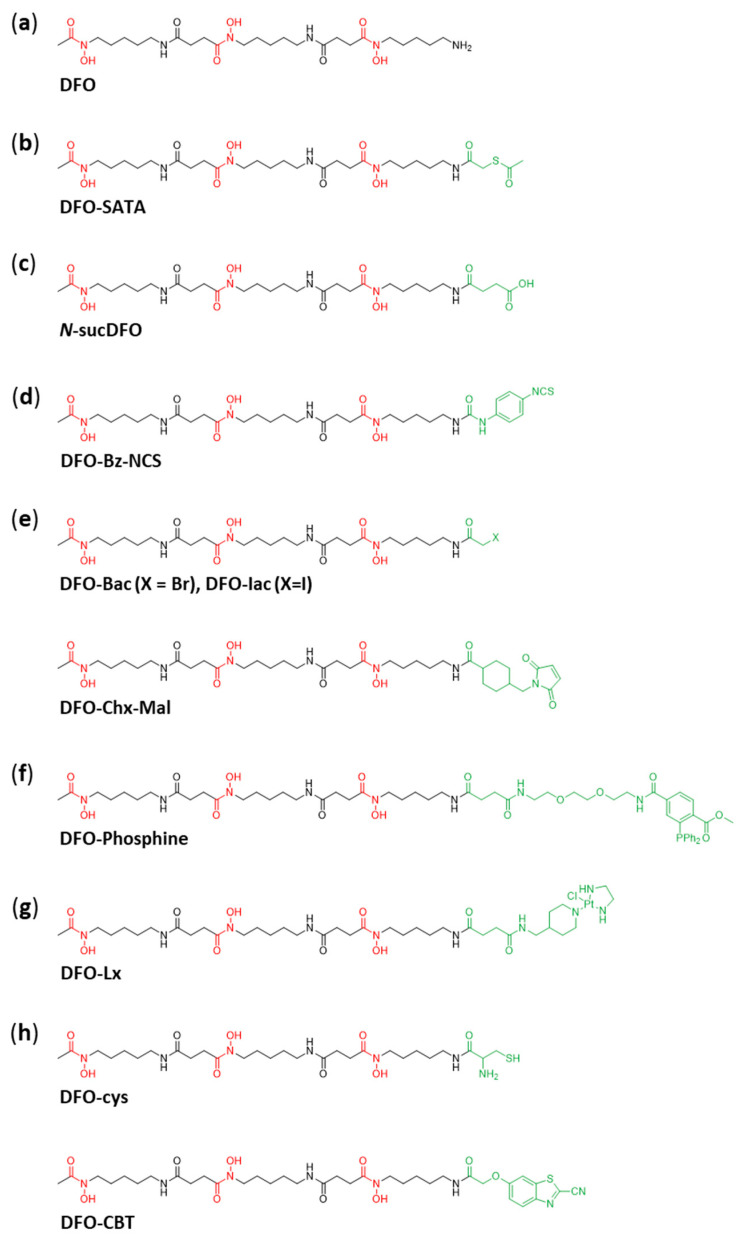
DFO and its bifunctional variants with the bifunctional moieties highlighted in green. (**a**) The chelator desferrioxamine (DFO) without bifunctionalization. (**b**) The first bifunctional variant of DFO, *N*-(*S*-acetyl)mercaptoacetyldesferal (DFO-SATA). (**c**) *N*-sucDFO was the first bifunctionalized DFO-chelator, that was used widely for ^89^Zr-radiolabeling of mAbs. (**d**) DFO-Bz-NCS allows the radiolabeling of mAbs in only two steps. (**e**) Three thiol reactive chelators; DFO-Bac, DFO-Iac and DFO-Chx-Mal. (**f**) DFO-Phosphine was developed for a pretargeting approach. (**g**) DFO-Lx is a bifunctionalized DFO variant using a platinum(II) linker. (**h**) For mAb-conjugation via click reaction through a luciferin-mediated approach DFO-cys and DFO-CBT were developed.

Returning to hydroxamate-based chelators, the focus of this review, different families of hydroxamic acids can be found in Nature. Their role in bacteria, fungi and plants is most commonly to solubilize and sequester iron (Fe^3+^) from the environment as siderophore (greek: “iron carrier”). The iron complex allows for delivery of the metal into the cell [22]. Hydroxamic acids were first chemically described by Heinrich Lossen in 1869 [23]. They provide excellent complexation properties for a large variety of metal ions, such as Fe^3+^, Mn^2+^ and Zr^4+^. Each hydroxamate group binds to the metal in a bidentate fashion via *O*,*O*-coordination to form stable five-membered ring structures (Figure 1) [24]. The only chelator used to date in the clinic to complex ^89^Zr for immunoPET is desferrioxamine (deferoxamine, DFO, Figure 3a). Due to its historical use in clinics to bind and remove excess of iron in blood (listed as essential medicine by the WHO) [25], its introduction into the clinic for immunoPET was obvious. However, recent preclinical studies indicate that the ^89^Zr-DFO complex lacks stability in vivo. This was concluded after the observation that unspecific accumulation of the ^89^Zr in bone tissue increased at late time points after injection of a ^89^Zr-radiolabeled compound. The free osteophilic zirconium concentrates into phosphate-rich hydroxyapatite bone matrix [26]. Such undesired uptake can compromise the detection of bone metastasis due to higher background signal but can also lead to an increased radiation dose to the bone marrow. Subsequent studies revealed that the hexadentate DFO with only three hydroxamate metal binding moieties does not saturate the coordination sphere of ^89^Zr. The favored octadentate coordination state of the ^89^Zr-DFO complex is therefore achieved in aqueous media by two less tightly bound water molecules [27,28,29]. The so created vulnerable coordination sphere is believed to be the reason for the complex instability over time in vivo. Interestingly, the increased accumulation of ^89^Zr in bones at late time points of immunoPET has, to date, only been witnessed in preclinical studies. Further clinical studies to investigate the mentioned phenomena are still pending.

In the following section, we are presenting the development and study of new DFO derivatives and other hydroxamate-based chelators for immunoPET using ^89^Zr since the first report in 1992 until recent developments in late 2020.

The initial publication by Meijs et al., which used the long lived ^88^Zr as an alternative to ^89^Zr, reported the use of DFO as a ^88^Zr-chelator and showed that DFO and ^88^Zr bound in a 1:1 stoichiometry with complexation being achieved rapidly at a wide pH range (pH 4–7) and a RCY of 95% [2]. They also demonstrated a high stability of ^88^Zr-DFO in plasma solutions with less than 0.2% of ^89^Zr released after 24 h.

Although DFO is not without its drawbacks, that has not stopped the development of a range of bifunctional chelators, based upon DFO, for use in immunoPET imaging. The first of these was *N*-(*S*-acetyl)mercaptoacetyldesferal (DFO-SATA; Figure 3b), reported by Meijs et al., which uses a thioester bond to couple DFO to albumins via its free Cys-34 residue [30]. The DFO-SATA bifunctional derivative showed rapid ^88^Zr labelling (RCY of >90% after 1 h) and high stability (<0.5% ^88^Zr demetallation over 24 h) during in vitro studies. Subsequently, biodistribution studies were carried out with ^88^Zr-citrate, ^89^Zr-DFO and ^88^Zr-labelled mouse albumin (^88^Zr-DFO-MSA). It was found that ^88^Zr-citrate rapidly accumulated in bones while the ^88^Zr-DFO was readily cleared from the body via the kidneys. The ^88^Zr-DFO-MSA on the other hand had a very similar biodistribution pattern as that of ^123^I-MSA, differing only in that there was a higher build-up of ^88^Zr in the liver, kidney and spleen. The lack of large amounts of ^88^Zr in the bones also suggested that the ^88^Zr-DFO-MSA complex was relatively stable in vivo. 

Despite these initial promising results, subsequent work by van Dongen and co-workers found that ^89^Zr-labelled mAb-DFO-SATA decomposed in vivo, due to instability of the thioether bond. Further experiments led to the conclusion that a ^89^Zr-chelate fragment transfers in vivo to serum proteins [31]. In the same article they reported a more stable DFO derivative (*N*-sucDFO; Figure 3c) which is linked to the mAb via a stable amide linker. Using this novel bifunctional chelator, they developed a protocol for the conjugation of mAb and subsequent ^89^Zr-labelling. The synthesis proceeds through a 2,3,5,6-tetrafluorophenol (TFP) ester of *N*-sucDFO. As such, before addition of the TFP ester, the hydroxamate groups of DFO had to be temporarily protected by chelating Fe(III). Following binding to the mAb, the complexed Fe(III) was removed with EDTA at pH 4.2–4.5 and chelation to ^89^Zr could be achieved. TFP-*N*-sucDFO is nowadays commercially available (ABX). The ^89^Zr-DFOsuc-*N*-mAb complex was then shown to give specific targeting and excellent detection of head and neck tumors in HNX-OE–bearing nude mice with a PET camera.

Several years later Perk et al. developed DFO-Bz-NCS (Figure 3d) [32]. This chelator binds to mAb through a stable thiourea bond and required only two-steps compared to the three-steps required for the labelling of mAb with *N*-sucDFO. This novel chelator, DFO-Bz-NCS, was covalently bound to three different mAbs and they found that the chelatore:mAb substitution ratio was consistently around 1.5:1 when using 3-fold molar excess of DFO-Bz-NCS. The radiolabeling of DFO-Bz-NCS-mAb could be achieved in HEPES buffer at pH 6.8–7.2 after 1 h with a RCY of >95%. A study comparing the stability of ^89^Zr-DFO-Bz-NCS-mAb and ^89^Zr-*N*-sucDFO-mAb found that ^89^Zr -DFO-Bz-NCS-mAb showed reduced radioconjugate stability if stored in the presence of chloride (Cl^−^) ions. This instability was proposed to arise from the radiation-induced formation of OCl^−^ ions which would then react with the enolised thiourea unit. This would subsequently lead to the formation of reactive sulphenyl chloride bonds which are known to undergo a variety of further reactions. The in vitro stability of both conjugates was shown to be comparable, with less than 4.7% release after 7 days of storage in human serum. Similarly, when the biodistribution and PET imaging data were compared for the two conjugates, a similar accumulation of ^89^Zr in organs and tissues was found in both cases.

In the same year Tinianow et al. published their work of three thiol reactive derivatives of DFO (DFO-Bac, DFO-Iac, DFO-Chx-Mal, Figure 3e) for site-specific mAb conjugation. Bioconjugation to thiotrastuzumab (mAb with engineered cysteine residues) was successful for all chelators and radiolabeling with ^89^Zr (1 h, RT, neutral pH) was achieved in yields greater 75%. Those ^89^Zr-chelator-mAb conjugates were found to be stable in mouse serum at 37 °C up to 96 h with no significant loss of radiometal. Furthermore, comparable PET-CT images (up to 144 h p.i.) were obtained in analogy to lysine conjugates of *N*-sucDFO and DFO-Bz-NCS as control (random conjugation). Beige nude XID mice, bearing BT474M1 xenografts, showed accumulation of 20–25% ID/g in tumor. Bone uptake was only significantly higher (*p* > 0.05) for ^89^Zr-DFO-Chx-Mal-thio-trastuzumab compared to the control conjugates [33].

A rather new approach in immunoPET used to reduce radiation dose to patients due to the slow pharmacokinetics of mAbs is pretargeting. Vugts et al. designed bifunctional DFO-phosphine derivatives (Figure 3f) which are able to undergo a biorthogonal Staudinger ligation to an azide-modified mAb (U36-triazide) in vivo. Radiolabeling of the DFO-phosphine derivatives was performed under standard conditions (2 h, RT, neutral pH) and achieved high yields (>93%). FastBlood clearance of the ^89^Zr-DFO-phosphines from tumor-free nude mice via gastrointestinal and urinary tract was observed during in vivo studies. The in vitro formation of Staudinger ligation in PBS between DFO-phosphine and the modified mAb also showed promising results. However, subsequent in vivo studies revealed reduced efficacy of Staudinger ligation in animals. After these experiments, the authors doubt the potential of the Staudinger ligation for pretargeting using the combination of phosphine-modified radiotracers with azide-modified mAbs [34].

Another bifunctional DFO derivative was developed using a platinum(II) linker [35]. Ethylenediamine platinum(II) was attached to DFO via linker (DFO-Lx, Figure 3g). Bioconjugation to trastuzumab was performed at 37 °C for 24 h. For radiolabeling studies Sijbrandi et al. used the protocol as previously described in the same group for *N*-sucDFO. Serum stability assays at 37 °C showed no release of ^89^Zr^4+^ over 200 h. To assess in vivo stability biodistribution studies were performed (72 h and 96 h) using nude mice bearing NCI-N87 xenografts comparing ^89^Zr-DFO-Lx-trastuzumab with the lysine coupled ^89^Zr-DFO-trastuzumab as control. Except of a slightly higher accumulation in liver for the Lx-conjugate, a very similar overall pharmacokinetic could be observed, including accumulation in bones.

More recently, an article by Gao et al. described the synthesis of two bifunctional DFO-based chelators which were attached to RGD peptides via a click reaction through a luciferin-mediated approach [36]. The synthesis of DFO-cys and DFO-CBT (Figure 3h) could be readily achieved and subsequent modification of RGD peptides with the orthogonal functionality allowed for the rapid preparation of the corresponding conjugates at 37 °C and pH 7.4. The conjugates could then be quantitatively labeled at RT after 90 min in HEPES buffer and both showed good in vitro stability.

## 4. DFO Derivatives

**Figure 4:**
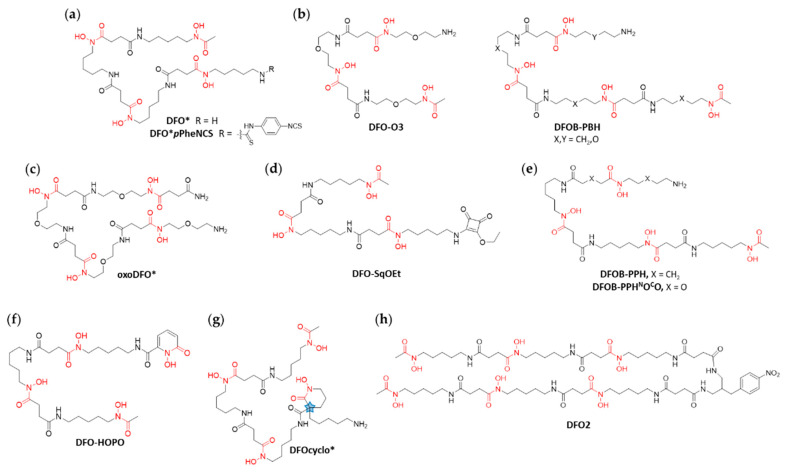
Structures of DFO derivatives. Additional hydroxamate moieties, compared to the three in DFO, lead to more stable metal-chelator complexes. (**a**) The octadentate DFO* extends the chain of DFO by one unit. DFO*pPheNCS is a bifunctionalized variant. (**b**,**c**) DFO-O3, DFOB-PBH and oxoDFO* contain oxygen in their backbones to increase hydrophilicity. (**d**) DFO-SqOEt uses a squaramide ester for bioconjugation chemistry and to increase its denticity. (**e**) DFOB-PPH and DFOB-PPH^N^O^C^O are linear tetrameric octadentate chelators. (**f**) DFO-HOPO is using a hydroxypyridinone moiety to extend the hexadentate DFO structure to an octadentate. (**g**) DFOcyclo* adds a cyclic hydroxamate motif to increase denticity. The blue star highlights the racemic center of the molecule. (**h**) DFO2 is a dimer of DFO ligands tethered together by a bifunctional linker.

Due to the excellent binding affinity of hydroxamate ligands to zirconium, ligands based on DFO with additional hydroxamate functionalities represent a promising improvement of the chelation of zirconium. Such an approach yielded the octadentate chelator termed DFO* (Figure 4a), which was reported by the groups of Mindt and Gasser for the first time in 2014 [37]. The structure was optimized by density functional theory (DFT) calculations and the synthesis of the compound was performed efficiently from commercially available DFO mesylate salt. The non-radioactive Zr-complex was evaluated by MS and NMR and a 1:1 Zr to ligand ratio was confirmed. DFO* as well as its bifunctional derivative DFO*-CO_2_H were successfully radiolabeled at pH 7.4 and RT with ^89^Zr-oxalate in quantitative yield. To evaluate the stability of the octadentate chelator, DFO*-CO_2_H was conjugated to the gastrin-releasing peptide receptor (GRPR) agonist [Nle^14^]BBS(7–14), the modified minimum binding sequence of bombesin (BBS), radiolabeled with ^89^Zr and compared with ^89^Zr-DFO-[Nle^14^]BBS(7–14) in challenging experiments using an excess of unconjugated DFO. The challenging experiments revealed a remarkably improved stability of ^89^Zr-DFO*-[Nle^14^]BBS(7–14) in comparison to the DFO complex (≈ 90% intact vs. <5% intact in 3000 fold excess of DFO after 24 h). In vitro binding assays with GRPR expressing PC3 cells were performed with the same compounds. Both exhibited comparable binding and internalization behavior, thus ruling out any impact of the chelators on the biological properties of the peptide [37].

Preclinical in vivo-results of DFO*-*p*PheNCS, conjugated to trastuzumab were published three years later in 2017 by Vugts et al. [38]. In this comprehensive study, the stability of both immunoconjugates ^89^Zr-DFO*-trastuzumab and ^89^Zr-DFO-trastuzumab was compared in vitro and in vivo. As before, the stability of ^89^Zr-DFO*-trastuzumab was shown to be much higher than that of the corresponding ^89^Zr-DFO conjugate in serum and NaCl solution (After 72 h at 37 °C, 97.5% and 77.7% intact tracer was left, respectively). In vivo PET imaging and biodistribution experiments in mice with N87 xenografts over 72 h revealed a comparable tumor uptake (47.7% ID/g for the DFO* conjugate vs. 35.5% ID/g for the DFO conjugate after 72 h) and blood clearance of the two immunoconjugates but significantly decreased uptake in bones for the octadentate chelator (1.7% ID/g vs. 3.0% ID/g in the sternum after 72 h, to give an example). The authors concluded that DFO*, which has become commercially available in the meantime, would be a worthy successor for DFO in clinical immunoPET [38].

The low solubility of DFO* in most solvents including water could represent a disadvantage regarding its application together with sensitive proteins without tolerance for DMSO or other co-solvents [39]. Therefore, the scaffold of DFO* was modified with oxygen atoms to increase its overall polarity and therefore water solubility. This approach was pursued independently by two different groups: (i) Richardson-Sanchez et al. developed a bioengineering route to synthesize the DFO derivative DFO-O3 (Figure 4b) with three oxygen atoms in its backbone [40]. The oxygen-containing backbone was generated from cultures of *Streptomyces pilosus* before the compound was extended with an additional hydroxamate group by synthetic methods to yield octadentate DFO* derivatives with increased water solubility (DFOB-PBH-O_1,2,3_, Figure 4b). However, the combination of bioengineering and chemical synthesis yielded the chelators only in limited amounts; (ii) the groups of Mindt and Gasser published a solid-phase assisted approach to yield a very similar octadentate hydroxamate chelator termed oxoDFO* (Figure 4c) with four oxygen atoms in its backbone [41]. LogD measurements performed with the free ligand as well as its Zr-complex showed increased water solubility in comparison to DFO*. Furthermore, the highly efficient synthesis would allow for the supply of the water-soluble derivative oxoDFO* on a gram scale. In the same study, an isothiocyanate containing bifunctional chelating agent (BFCA) of oxoDFO* for fast and efficient conjugation to proteins was developed [41].

^89^Zr-radiolabeling and stability studies with oxoDFO* were published three years later in 2020 by the same groups [42]. Quantitative conversion of the water-soluble chelator to the desired complex ^89^Zr-oxoDFO* was achieved (2 h, RT, pH 7.4), which are conditions applicable to sensitive Abs. In challenging experiments to compare in vitro stabilities, the ^89^Zr-complexes of octadentate DFO* and oxoDFO* clearly outperformed ^89^Zr-DFO due to their higher denticity (90% intact vs. >20% intact in 50 mM DTPA solution after 48 h) [42]. To date, in vivo data of the oxygenated analogs of DFO*, oxoDFO*, are still awaited.

A DFO derivative equipped with a squaramide ester, termed DFOsqOEt (Figure 4d), was investigated and evaluated in vitro and in vivo by the Donnelly group [43]. By using a squaramide ester for bioconjugation chemistry, the authors expected an octadentate coordination to ^89^Zr due to the presence of the additional functionalities. Stability studies in presence of excess EDTA showed that the complex ^89^Zr-DFOsqOEt was more resistant to ligand exchange than previously used ^89^Zr-DFO (82% intact vs. 70% intact in 50 mM EDTA at 50 °C, 24 h). Conjugated to the mAb trastuzumab and radiolabeled with ^89^Zr, the chelator was used for PET imaging in a tumor mouse model. Interestingly, the tumor-to-bone ratio of ^89^Zr-DFOsqOEt-trastuzumab was increased by a factor 2 in comparison to the DFO immunoconjugate (9 vs. 4 after 48 h, respectively). In ex vivo biodistribution experiments, a substantial spleen uptake (around 100% ID/g) was detected for ^89^Zr-DFOsqOEt-trastuzumab.

The most comprehensive in vivo comparison of the chelators DFO-NCS, DFO*-NCS, DFOsqOEt and DFO*sqOEt so far was reported by Chomet et al. [44]. The study not only revealed important insights into in vivo stability of the ^89^Zr-complexes, but also provided valuable in vitro stability data, which contribute to the understanding of the complexation behavior of the squaramide group. The chelators were conjugated to cetuximab, trastuzumab and a non-binding control mAb and subsequently radiolabeled with ^89^Zr. The stability of the conjugates was evaluated not only in serum, but also in an excess of multiple challenging ligands in different concentrations as well as in the presence of other metal cations. In all cases, ^89^Zr-DFO* derived conjugates resulted in higher stability compared to their DFO counterparts, with only a slightly improved stability of ^89^Zr-DFOsqOEt-mAb in comparison to ^89^Zr-DFO-*p*PheNCS-mAb. The significant difference between the stabilities of DFOsqOEt and the DFO* derivatives substantiates the suspicion that the squaramide group is not coordinating the central ion, or not with the same strength as the hydroxamates. The results of this study once again underline the superiority of octadentate chelators for zirconium.

Preclinical in vivo studies were performed in multiple tumor models and particularly a bone metastasis model to assess the bone uptake and the tumor-to-bone-ratio in vivo. In line with the in vitro results, the ^89^Zr-DFO*pPheNCS and ^89^Zr-DFO*-SqOEt labeled mAbs outperformed their DFO and DFOsqOEt counterparts in terms of a drastically reduced bone uptake (knee uptake: 1.2% ID/g for ^89^Zr-DFO*-*p*PheNCS-trastuzumab, 1.6% ID/g for ^89^Zr-DFO*-SqOEt-trastuzumab, 7.9% ID/g for ^89^Zr-DFO-*p*PheNCS-trastuzumab and 12.1% ID/g for ^89^Zr-DFOsqOEt-trastuzumab 144 h p.i.), while the tumor uptake and overall in vivo behavior remained the same. Additionally, in vivo studies on the intratibial BT-474 model showed much better tumor-to-background contrast due to less unspecific accumulation of ^89^Zr in bones. The authors concluded that DFO-immunoconjugates are less suitable for the detection of bone metastases and again highlighted the superiority of DFO* derivatives in comparison to the current gold standard DFO [44].

Similar results were found in a study involving rhesus monkeys for up to 30 days after injection of ^89^Zr-radiolabeled immunoconjugates using DFO-NCS, DFO*-*p*PheNCS, DFOsqOEt and DFO*sqOEt published by Berg et al. [45]. The comparative study using humanized monoclonal IgG conjugates was conducted to evaluate the pharmacokinetic behavior especially at later time points. As a result, the ^89^Zr-labeled complexes of DFO*and DFO*-SqOEt, conjugated to an Ab against the herpes simplex viral protein glycoprotein D (anti-gD) showed remarkably less (a third to a quarter) bone uptake 30 days p.i. in comparison to DFO. In contrast to that, ^89^Zr-DFOsqOEt-anti-gD showed not only substantial uptake in bones and liver, but also slower clearance from the body (≈65% ID/g after 30 days vs. <40% for ^89^Zr-DFO*sqOEt-anti-gD, ^89^Zr-DFO-pPhe-NCS-anti-gD and ^89^Zr-DFO*-*p*PheNCS-anti-gD). Overall, consistent data as well as excellent imaging quality was achieved during this study, revealing important facts about the fate of radiolabeled mAb conjugates for time points up to nine half-lives of ^89^Zr p.i. [45].

Another addition to the family of DFO-derivatives with modified backbones is represented by DFOB-PPH, and DFOB-PPH^N^O^C^O (Figure 4e), two linear tetrameric octadentate chelators [46]. The PPH derivative resembles DFO* (surprisingly renamed into DFOB-PBH in the publication by Brown et al.). The structure possesses an additional methylene group, which is postulated to result in higher complex stability due to the increased volume of the coordination sphere. In competition studies with EDTA, it was shown that the Zr-complexes of DFO* and DFOB-PPH show comparable stability. The publication also contained a synthetic route to an isothiocyanate BFCA derivative of DFOB-PPH, as well as the oxygen-containing, water soluble version DFOB-PPH^N^O^C^O. In a competition experiment of DFOB-PPH^N^O^C^O in the presence of Fe^+3^ and Zr^+4^, the formation of the Zr-complex of DFOB-PPH^N^O^C^O was favored towards the iron complex due to entropic effects, whereas its stability towards transchelation in excess EDTA was lower than the stability of Zr-DFOB-PPH and Zr-DFO* (≈50% intact vs. ≈80% for DFOB-PPH and DFO*, respectively in 1600-fold excess of EDTA after 24 h) [46].

The following two publications of DFO-derived chelators extend DFO by a cyclic hydroxamic acid to achieve octadentate chelators [47,48]. The concept of applying cyclic hydroxamic acids for metal complexation was previously described by Jewula et al. [49]. Acyclic hydroxamate acids show limited rotation around the central C–N bond and exist therefore preferred in the *trans* form. Cyclic hydroxamic acids on the other hand are preorganized in the cis form, ideal for metal chelation.

The structure of DFO-HOPO (Figure 4f) was already described in 1988 by White et al., yet not for the complexation of ^89^Zr but for the treatment of plutonium(IV) poisoning [50]. DFO-HOPO, however, delivers the required structural properties as an octadentate chelator for ^89^Zr. Hence, in 2017, Allott and Pieve et al. reported an optimized synthesis of DFO-HOPO and its evaluation for ^89^Zr chelation [47]. DFT analysis showed that the metal center coordinated to eight oxygen atoms of DFO-HOPO. Quantitative radiolabeling could be achieved in comparable efficiency to DFO. Stability experiments were performed by a challenge assay at pH 7 with 100-fold molar excess of EDTA over seven days. ^89^Zr-DFO-HOPO showed no transchelation, whereas ^89^Zr-DFO lost about 35% of the metal ion. In vivo studies by PET imaging revealed renal clearance with high radioactivity in the bladder 1 h p.i. At later timepoints, however, hepatobiliary excretion with high uptake in the gut 4 h and 24 h p.i. was observed, which was confirmed in biodistribution experiments. Furthermore, accumulation in bones was monitored and found to be 10-fold higher for ^89^Zr-DFO at 24 h p.i. compared to ^89^Zr-DFO-HOPO (0.04% ID/g and 0.004% ID/g, respectively). The results for this novel ^89^Zr-chelator are promising, yet bifunctionalization and bioconjugation are still pending.

Raavé et al. developed another octadentate DFO derivative with a cyclic hydroxamate motif, DFOcyclo* (Figure 4g) [48]. The evaluation of DFOcyclo* was performed in parallel to comparison studies with DFO and DFO*. Stability assays in EDTA solution (1000-fold molar excess) of ^89^Zr-DFO, ^89^Zr-DFO* and ^89^Zr-DFOcyclo* over seven days confirmed the low stability of ^89^Zr-DFO (53% intact), but similarly excellent stability for ^89^Zr-DFO* and ^89^Zr-DFOcyclo* (>98%). The novel chelator was bifunctionalized with an isothiocyanate group and conjugated to the Ab trastuzumab. Subsequently, in vivo studies of the conjugate were performed in comparison to analog conjugates of DFO and DFO* in nude mice bearing HER2^+^ SKOV-3 xenografts. Blood levels and accumulation in tumors over 168 h p.i. were similar for the three radiotracers. However, uptake of radioactivity in bones was significantly lower for ^89^Zr-DFOcyclo*-trastuzumab and ^89^Zr-DFO*-trastuzumab in comparison to ^89^Zr-DFO-trastuzumab. These results demonstrate interesting properties for the novel chelator DFOcyclo*, however, the racemic structure of the compound might be a challenge when moving towards clinical applications.

An interesting study was reported in the same article of Raavé et al. to address the question about the mechanism of the release of ^89^Zr in vivo that ultimately leads to its unspecific accumulation in bones. One hypothesis that has been reported is that zirconium is released after internalization and intratumoral metabolism [27]. To investigate this, a comparison of the metal release and subsequent accumulation in bone was carried out between the above described HER2-positive tumor bearing mice and mice with HER2-negative tumor xenografts. A confirmation of the hypothesis would be a lower bone uptake in the latter case. However, no significant difference could be found between the groups, rebutting the hypothesis.

The latest addition to the collection of novel DFO-based chelators was reported by Sarbisheh et al. in 2020. DFO2 is a dimer of DFO ligands tethered together by a bifunctional linker. The resulting chelator has six hydroxamic acid groups and therefore a potential coordination number of 12 (Figure 4h) [51]. DFT analysis of the ^89^Zr-DFO2 complex suggested an octadentate structure. However, due to the additional two hydroxamates, over 500 geometric isomers are principally possible. The chelator, with a *p*-NO_2_-Ph moiety for later bifunctionalization, showed very low water solubility, which could be a potential issue for bioconjugation chemistry. Quantitative radiolabeling was achieved at pH 6.8−7.2, 60 min, 37 °C. The two complexes (^89^Zr-DFO2 and ^89^Zr-DFO) were incubated with a hydroxyapatite resin. ^89^Zr-DFO2 showed superior stability after 24 h compared to ^89^Zr-DFO. This was also confirmed by EDTA challenge assays (100-fold molar excess) at different pH (pH 5–8) over seven days. As in the case for other new chelators discussed in this review, data on their performance in vivo is still pending.

## 5. Not-DFO-Based Hydroxamate Chelators

**Figure 5:**
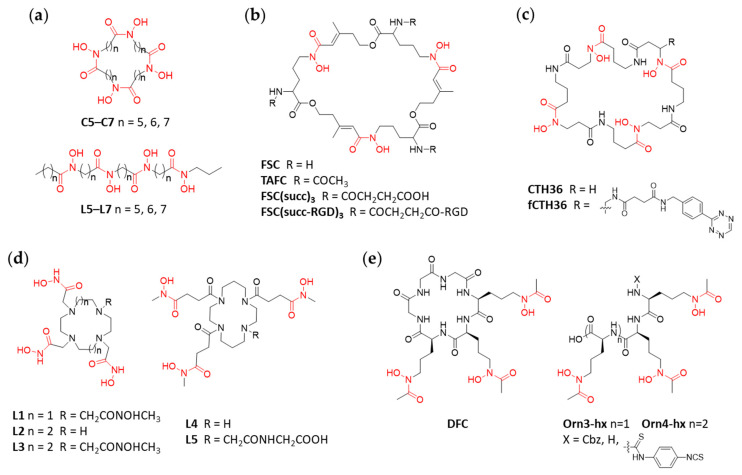
Structures for cyclic not-DFO-based hydroxamate ^89^Zr-chelators and linear derivatives. (**a**) Three macrocycles C5–C7 and their acyclic analogs L5–L7. (**b**) The macrocyclic Fusarinine C (FSC) and derivatives. (**c**) A Cyclic Tetra-Hydroxamate (CTH) 36-membered macrocycle. (**d**) Cyclen- (L1–L3) and cyclam- (L4, L5) based macrocycles. (**e**) The macrocyclic desferrichrome (DFC) and its linear derivatives Orn3-hx/Orn4-hx with three or four hydroxamates, respectively.

After discussing hydroxamate chelators based on the structure of DFO, in the following sections we will focus on other scaffolds investigated for hydroxamate chelators for ^89^Zr. In 2014 Guérard et al. reported a set of new macrocyclic hydroxamate-based chelators for applications in ^89^Zr-radiotracer development, the design of which was based on X-ray structure analysis. The examples included four hydroxamate groups separated by spacer units of different length including five, six or seven carbon atoms (C5, C6, C7; Figure 5a). In parallel, and for comparison, the synthesis and evaluation of their acyclic analogs (L5, L6, L7) was also included in the study [52]. The high lipophilicity and therefore insolubility of the compounds in aqueous media was identified as a challenge as we and others have also experienced [37,42]. While RCY >95% could be achieved at RT (pH 7, 120 min) with the ligands having longer spacers (C7, L6, L7), elevated reaction temperature was required for the ^89^Zr-labeling of the shorter analogs (C5, C6, L5). Stability studies (phosphate buffer, pH 6.5) and transchelation experiments (50 mM EDTA, pH 7, 37 °C) showed that only ^89^Zr-C7 and ^89^Zr-L7 outperformed ^89^Zr-DFO. In summary, this work demonstrated that a fourth hydroxamate group together with the macrocyclic effect can significantly improve the stability of ^89^Zr-complexes. No biological data is yet available for the reported chelators.

An initial study of Decristoforo and co-workers compared selected siderophores as ^68^Ga and ^89^Zr-chelators [53]. One of such that has been investigated in the context of ^89^Zr-radiolabeling is fusarinine C (FSC; Figure 5b). With three hydroxamate moieties, FSC is structurally similar to DFO but differs in that it is a cyclic compound. Additionally, FSC contains three primary amines, which allow for further functionalization, e.g., to increase its denticity or to form multimers when biological vectors are conjugated to it. In 2015, Zhai et al. reported on the ^89^Zr-labeling of triacetyl fusarinine C (TAFC) and tumor targeting trimer FSC(succ-RGD)_3_ with RGD being a small cyclic peptide [54]. In 2019, further examples including FSC(succ)_2_Ac and FSC(succ)_3_ followed (Ac = acetate, succ = −COCH_2_CH_2_COOH) which expanded the hexadentate FSC to an octa- and potentially nonadentate chelator able to saturate the coordination sphere of Zr^4+^ [55]. Radiolabeling of the novel chelators with ^89^Zr-oxalate was achieved in quantitative yields at RT and neutral pH. Assessment of the ^89^Zr-complexes and conjugates by transchelation experiments in the presence of 1000-fold excess EDTA at different pH revealed that all FSC-based chelators performed better than DFO with the highest stability for ^89^Zr-TAFC and ^89^Zr-FSC(succ)_3_ (e.g., at pH 7 ^89^Zr-DFO lost close to 60% of the metal ion, where the next weakest ligand ^89^Zr-FSC(succ-RGD)_3_ lost only 4%.) [54,55]. As seen in previously discussed examples, this can be ascribed to both the proper denticity of the chelator and the macrocyclic effect. ^89^Zr-FSC(succ-RGD)_3_ was also evaluated in female, athymic BALB/c nude mice by biodistribution and quantitative PET/CT imaging experiments. The radiotracer showed high specificity towards its target, α_v_β_3_ integrin, and fast pharmacokinetics with predominant renal excretion. Uptake of radioactivity in the bones was <1% ID/g at 4 h p.i. indicating good stability in vivo [54], however, one has to acknowledge that this time point may be too early for determining in vivo stability as unspecific bone uptake of ^89^Zr is usually highest after several days in the case of ^89^Zr-labellebed Ab. Biodistribution studies in healthy mice were performed for ^89^Zr-TAFC and ^89^Zr-FSC(succ)_3_. Fast pharmacokinetics with predominant renal excretion was observed. The results were confirmed by PET/CT imaging [55].

The Cristoforo group continued their work with ^89^Zr-FSC radiotracers by conjugating a maleimide-functionalized FSC to an EGFR targeting affibody (Z_EGFR:2377_) via a C-terminal cysteine residue [56]. ^89^Zr-radiolabeling of FSC-Z_EGFR:2377_ was performed at neutral pH and both at 28 °C or 85 °C. It was observed, unlike in the case of experiments with the chelator alone, that the efficiency of the formation of the ^89^Zr-complex was significantly slower at RT but could be increased at higher temperature. Subsequent studies on the stability of ^89^Zr-FSC-Z_EGFR:2377_ by transchelation experiments in the presence of 1000-fold excess EDTA (in PBS) revealed that the complex formed at lower temperature surprisingly exhibited a lower stability than the product obtained at 85 °C (at 24 h 97% vs. 85%, respectively). This was not the case for the corresponding ^89^Zr-DFO-Z_EGFR:2377_ which served as a reference compound. The authors concluded that this may be the result of a weak unspecific coordination of ^89^Zr by FSC-Z_EGFR:2377_ at RT. Regardless, the ^89^Zr-FSC-conjugates displayed overall an improved in vitro stability in comparison to the analog ^89^Zr-DFO compound. Subsequent biodistribution studies (3 h and 24 h p.i.) with ^89^Zr-FSC-Z_EGFR:2377_ in female BALB/c ^nu/nu^ mice bearing A431 tumor xenografts, were performed in a direct comparison with ^89^Zr-DFO-Z_EGFR:2377_. The ^89^Zr-FSC-conjugate revealed the typical affibody elimination via renal pathway but exhibited a 1.5-fold higher tumor uptake than DFO-conjugates (3 h p.i., 8.4% ID/g vs. 5.9% ID/g). Also, a significantly higher accumulation in the liver was found. The unspecific uptake of radioactivity in bones is reported to be ∼1% ID/g 24 h p.i. for both, ^89^Zr-DFO-Z_EGFR:2377_ and ^89^Zr-FSC-Z_EGFR:2377_. However, for the DFO-conjugate an increase of accumulation in bones was found starting from 0.5% ID/g 3 h p.i., whereas the FSC-conjugate showed a steady value [56].

Seibold et al. used a computational approach for the development of yet another class of macrocyclic chelators bearing four hydroxamate moieties [57]. The first objective was to identify the proper cavity size of the cyclic chelator for matching the radius of the central ion Zr^4+^. The computational studies indicated that the optimum complex stability could be achieved with a 36 membered ring motif. Further DFT analysis confirmed a complete envelopment of Zr^4+^ by the octadentate ligand CTH36 (Cyclic Tetra-Hydroxamate; Figure 5c). Subsequently the chelator was prepared by both solid phase and in-solution syntheses of which the latter proved to be more successful. Bifunctionalization and conjugation to the model peptide c(RGDfK) was achieved via conversion of the pending amine group of the derivative fCTH36 into a tetrazine derivative followed by copper-free click chemistry (inverse electron demand Diels-Alder (IEDDA) with c(RGDfK) functionalized with a *trans*-cyclooctene [58]). Analogously, the corresponding DFO-c(RGDfK) conjugate was synthesized as a reference compound. Radiolabeling of both conjugates with ^89^Zr-oxalate was achieved in quantitative yields at RT and neutral pH. Transchelation experiments with ^89^Zr-CTH36-c(RGDfK) and ^89^Zr-DFO-c(RGDfK) in the presence of 100–9000-fold excess of EDTA at pH 7 demonstrated a higher stability of the ^89^Zr-CTH36 complexes. No biological data on ^89^Zr-labeled CTH36 derivatives have yet been reported.

Remarkably, the cavity size of the most successful macrocyclic chelators described in the publications above are all composed by a 36-membered ring with three or four hydroxamate groups. Their success is demonstrated by outmatching DFO in the ^89^Zr-complex stability. This shows the importance of the right cavity size in respect to the metal ion.

The universal cyclic scaffolds of cyclen and cyclam ligands have also been explored in the context of the development of ^89^Zr-chelators. Boros et al. reported on hexa- and octadentate hydroxamate versions of these scaffolds (L1–L4; Figure 5d) as well as a bifunctionalized version thereof for bioconjugation chemistry (L5) [59]. The ^89^Zr-radiolabeling of L1–L4 and the stability of the resulting radiometal complexes was assessed and compared to DFO. While the ^89^Zr-radiolabeling under standard conditions (pH 7.4–7.6, 1 h, RT) was equally efficient for all compounds, the in vitro stability of ^89^Zr-L1–L4 did not show any improvement in comparison to ^89^Zr-DFO as determined by challenging experiments with EDTA (50 mM, pH 7, 37 °C, 6 days). DFT calculations indicated that derivatives with short linkers (L1–L3) were not providing the opportunity for octa-coordinated ^89^Zr-species due to steric constrains. On the other hand, L4 appeared to have promising (hexadentate) chelation properties based on the computational analysis. Therefore, ^89^Zr-labeled L4 and its bifunctionalized version L5 conjugated to trastuzumab were further investigated in vivo. However, in comparison to the corresponding ^89^Zr-DFO reference compounds, no improvement in terms of tumor-targeting or unspecific uptake of radioactivity in bones could be confirmed. This example illustrates again the need of octadentate chelators for the stable complexation of ^89^Zr in vivo.

Desferrichrome (DFC; Figure 5e), a cyclic hexapeptide derived from ornithine, is a naturally occurring fungal siderophore. Adams et al. evaluated DFC as novel chelators for the complexation of ^89^Zr in immunoPET applications. In parallel, hexa- and octadentate linear versions of DFC, with three or four hydroxamate moieties (Orn3-hx and Orn4-hx, respectively; Figure 5e) were investigated and compared to DFO [60]. Quantitative radiolabeling with ^89^Zr-oxalate could be achieved for all ligands at RT and physiological pH. DFT analysis predicted the octadentate Orn4-hx to achieve the most stable complex with ^89^Zr among the ones described. Interestingly, the two hexadentates DFC and Orn3-hx showed seven coordinated complexes by including one water molecule (not two, as initially expected [27]). The computationally predicted complex stabilities were confirmed by transchelation challenge experiments with 1000-fold excess of EDTA. ^89^Zr-Orn4-hx and ^89^Zr-DFO showed comparable results, ahead of ^89^Zr-DFC and ^89^Zr-Orn3-hx (t = 2 h; 64%, 67%, 53%, 39%, respectively). For bioconjugation to the Ab trastuzumab, an isothiocyanate functionality was introduced into Orn-3hx and Orn-4hx. To evaluate the conjugates in vivo, naïve C57BL6 mice were injected intravenously with ^89^Zr-Orn-3hx-trastuzumab or ^89^Zr-Orn-4hx-trastuzumab for biodistribution studies (96 h p.i.) and compared to ^89^Zr-DFO-trastuzumab. Both desferrichrome conjugates showed significant faster blood clearance but also significantly higher liver uptake compared to the DFO-conjugate. The in vivo (naïve C57BL6 mice) stability of the three ^89^Zr-chelator-Ab complexes was evaluated and compared through the accumulation of radioactivity in bones by biodistribution studies. As found in the transchelation challenge experiments, ^89^Zr-Orn-4hx-trastuzumab and ^89^Zr-DFO-trastuzumab showed comparable results with slightly higher stability (bone uptake at 96 h p.i. 7% ID/g) compared to ^89^Zr-Orn-3hx-trastuzumab (bone uptake at 96 h p.i. 10% ID/g). The gathered results by Adams et al. show the potential of DFC derivatives with comparable stability of Orn-4hx to DFO.

**Figure 6:**
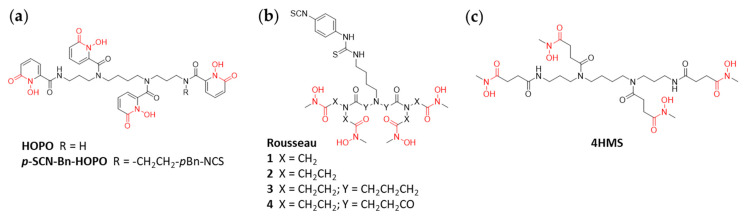
Acyclic hydroxamate chelators. (**a**) 1,2-hydroxypyridone based chelator. (**b**) symmetrical chelators (Rousseau 1 and 2) based on a 2,2′-iminodiacetamide backbone; Rousseau 3 and 4 based on extended iminodipropionamide and dipropylenetriamine backbones, respectively. (**c**) A spermine backbone-based chelator.

An early example of a rationally designed acyclic chelator with a backbone different to that of DFO is 3,4,3-(LI-1,2-HOPO) (HOPO; Figure 6a), which was first reported by Deri et al. in 2014 [61]. HOPO contains four hydroxypyridone moieties which allow formation of an octadentate complex with Zr^4+^. DFT calculations suggested that HOPO would provide more stable Zr^4+^ complexes than DFO, a theoretical finding which was later confirmed experimentally. Radiolabeling of HOPO with ^89^Zr could be achieved quantitatively after 1 h. Challenging experiments of ^89^Zr-HOPO and ^89^Zr-DFO in the presence of 100-fold excess EDTA at pH 5–8 showed a remarkable stability of the former complex even after 7 days whereas transchelation of ^89^Zr was observed for the latter reference complex at all pH investigated (e.g., pH 7.5, 100% vs. 76%). In subsequent PET imaging in healthy mice, it was found that ^89^Zr-HOPO cleared rapidly from the body via renal excretion and, more importantly, no accumulation of radioactivity was observed in the bones (up to 24 h p.i.) indicating that no release of ^89^Zr from the complex occurred in vivo within this timeframe. In a next step, a bifunctional variant of HOPO for bioconjugations was developed [62]. *p*-SCN-Bn-HOPO (Figure 6a) was conjugated to lysine residues of trastuzumab via thiourea bond formation as was DFO-*p*Bn-NCS to obtain a reference conjugate. Both Ab conjugates were readily radiolabeled with ^89^Zr (pH 7, ^89^Zr-oxalate, 1–3 h) in quantitative RCYs and showed high stability (>89% intact) in human serum over 7 days. However, significant differences between the two conjugates were observed in vivo by biodistribution experiments and PET imaging using female, athymic nude mice with BT474 breast cancer xenografts. The uptake of ^89^Zr-DFO-trastuzumab in the tumors was more than doubled in comparison to that of ^89^Zr-HOPO-trastuzumab (138.2% ID/g vs. 61.9% ID/g at 336 h). Unspecific uptake of ^89^Zr in bones was initially low for both ^89^Zr-conjugates, however, while it remained constant for 9 days in the case of ^89^Zr-HOPO-trastuzumab (2.5% ID/g), a steadily increasing accumulation of radioactivity in bones was observed for ^89^Zr-DFO-trastuzumab (up to 17.0% ID/g). This data suggests a steady leaching of the radiometal from ^89^Zr-DFO-trastuzumab. Finally, the low yield and lengthy synthesis of HOPO and its derivatives has been considered a major limitation for its wide-spread use. This concern has recently been resolved by the development of an optimized synthetic procedure that requires fewer steps and provides the desired compounds in improved yields [63].

Work by Rousseau et al. focused on the development of new symmetrical chelators based on a 2,2′-iminodiacetamide backbone (Figure 6b) [64,65]. Mainly, the chelators investigated differ from each other in terms of the length and chemical composition of the linker separating the hydroxamate moieties. The examples of Rousseau chelators with longer linkers provide more spatial and structural flexibility for the complexation of the radiometal. The chelators were readily synthesized, functionalized with a *p*Bn-NCS moiety, and conjugated to trastuzumab. In analogy, DFO-*p*Bn-NCS was conjugated to trastuzumab to obtain a reference compound. Radiolabeling of all conjugates with ^89^Zr under standard conditions (oxalic acid/sodium carbonate, pH 7–7.4, RT, 1 h) efficiently provided the corresponding radiolabeled conjugates (RCY > 90–95%), though the stabilities of these ^89^Zr-labelled Rousseau chelator conjugates were found inferior to that of the reference DFO-trastuzumab both in vitro and in vivo.

Recently, Alnahwi et al. reported on 4HMS, a novel octadentate chelator based on the scaffold spermine as used for the above discussed HOPO chelators (Figure 6c) [66]. The synthesis of 4HMS was straight forward as was its quantitative ^89^Zr-labeling (10 min, pH 7, RT, ^89^Zr-oxalate). In comparison to ^89^Zr-DFO, the achievable apparent molar activity was three times higher for ^89^Zr-4HMS (170 GBq/μmol). In challenging experiments with DTPA (100 equiv.), ^89^Zr-4HMS showed excellent stability (>95.5%) even after 7 days at all pH values tested (pH 5, 7, 8.5). The improved stability of ^89^Zr-4HMS in comparison to ^89^Zr-DFO was also confirmed by challenging the ^89^Zr-complexes in the presence of an excess of biologically relevant metal ions (iron, cobalt, copper, nickel, magnesium, calcium). Only in the case of 100 equivalents of iron (FeCl_3_), significant transmetallation was observed after 7 days (62% for ^89^Zr-4HMS vs. 38% for ^89^Zr-DFO). In vivo studies in healthy Balb/c mice compared ^89^Zr-4HMS to ^89^Zr-DFO at 1, 4 and 24 h time points. ^89^Zr was shown to accumulate in the kidneys at 1 h for both chelators, suggesting that ^89^Zr is rapidly cleared from the body via renal excretion. ^89^Zr-4HMS showed minimal accumulation of ^89^Zr in the bones at 24 h compared to ^89^Zr-DFO (0.01% ID/g vs. 0.17% ID/g) which also confirms the higher stability of the ^89^Zr-4HMS complex. 4HMS shows great promise as ^89^Zr-chelator with slightly better stability against biologically relevant ions compared to HOPO and comparable stability to DFO*. However, evaluations with bioconjugates are still to be done.

## 6. X-ray Structures and Thermodynamic Stability of ^89^Zr-Complexes

**Figure 7:**
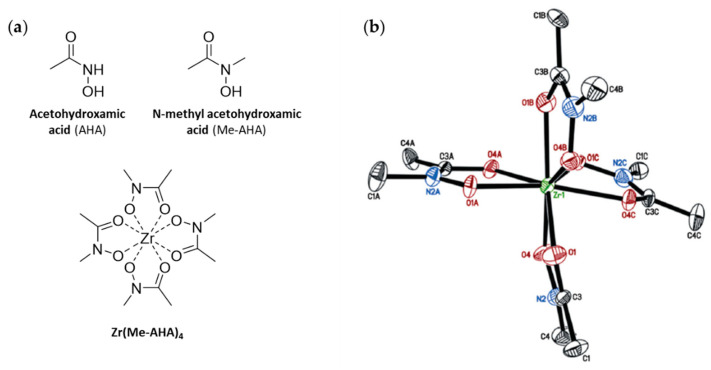
(**a**) Chemical structures of AHA, Me-AHA and Zr(Me-AHA)_4_. (**b**) X-ray structure of Zr(Me-AHA)_4_ obtained from the 0.572 × 0.258 × 0.097 mm^3^ crystal. Displacement ellipsoids are shown at the 50% level, hydrogen atoms and water molecules have been omitted for clarity. Reproduced from [68] with permission from The Royal Society of Chemistry (https://pubs.rsc.org/en/content/articlelanding/2013/cc/c2cc37549d, accessed on 3 September 2021).

Experimental and theoretical data on the mode of coordination and thermodynamic stability of Zr-complexes are scarce, likely due to the fact that Zr^4+^ ions are highly acidic and tend to hydrolyze in aqueous conditions, leading to insoluble Zr-oxide species [67]. Nonetheless, there have been several studies that have looked at trying to get a better insight and understanding of the complexation of Zr^4+^ by DFO through X-ray analysis, computational studies, and thermodynamic experiments. The first of these was published by Guérard et al. in 2013 [68]. Using acetohydroxamic acid (AHA) and *N*-methyl acetohydroxamic acid (AHA-Me) as simple hydroxamates, the authors were able to produce the first examples of X-ray crystal structures of a Zr-complex with AHA and AHA-Me (Figure 7). Via analysis of the X-ray crystal structures in combination with potentiometric studies it was shown that zirconium has a preference to be octadentate with each hydroxamate ligand binding through the two oxygen atoms to the central Zr^4+^ ion. The fact, that this publication was only recently discussed by a comment [69] and a subsequent reply [70] demonstrates the task of analyzing zirconium-hydroxamate complex formations remains challenging. Holland and Vasdev used the same model for in depth DFT investigations of the mechanism of ligand substitution between Zr-oxalate and MeAHA [71].

Building upon this work, Savastano et al. investigated the speciation of Zr^4+^-DFO complexes in water in 2019 [72]. Using a combination of potentiometric and small-angle X-ray scattering (SAXS) measurements, they were able to show that metal to ligand stoichiometry of the complexes formed varied from 1:1, 2:2 and 2:3 across a wide range of pH (pH 2.5–11.5). This range of stoichiometry was confirmed through MALDI-TOF/TOF spectrometry. The stability constants of all mono- and binuclear species present in solution were calculated and the log β value for [Zr-DFO]^+^ was found to be 36.14, which indicates a thermodynamically very stable complex.

In the same year that Savastano et al. published their work on the speciation of Zr-DFO complexes, Toporivska et al. reported on the thermodynamic stability of [Zr-DFO]^+^ (log β = 40.06) complexes in solution [73]. They used an array of techniques, including ESI-MS, potentiometry, UV-Vis spectroscopy and isothermal titration calorimetry, to determine both the stoichiometry of [Zr-DFO]^+^ complexes and their stability over a wide pH range (pH 1–11). Contrary to the discovery of Savastano et al., they proposed a model containing only mononuclear, 1:1, species across the pH range, and that the apparent instability of Zr^4+^-DFO in vivo was the result of the deprotonation of one of the water molecules from the outer sphere of the Zr^4+^. Both groups proposed the addition of an extra chelating group in the ligand and that the use of a cyclic ligand could lead to better in vivo stability.

**Figure 8:**
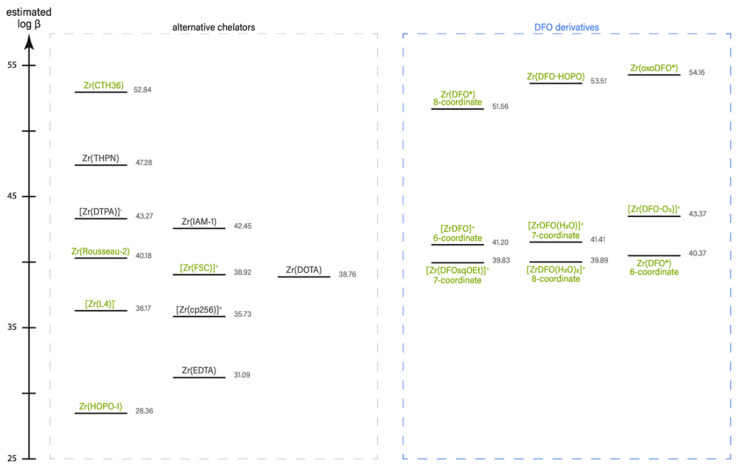
Plot of estimated formation constants for different Zr^4+^ complexes. Ligands derived from DFO are shown in the right panel, others are grouped in the left panel. Black font-color indicates non-hydroxamate chelators, green hydroxamate chelators. Figure adapted from [28].

A recent publication, that cannot be left aside in a review about zirconium chelators, is an article by Holland on the prediction of the thermodynamic stability of zirconium radiotracers [28]. In this work, DFT calculations were used to predict the reaction energetics for metal complexation, hence, the absolute and relative thermodynamic stability (expressed as the formation constant log β). Additionally, attention was paid to the effects of metal-aquo ion complexes, [M(H_2_O)n], by incorporating first, second, and third hydration shells to the metal ion. To verify the theoretical predictions additional experimental data were collected in parallel, which showed a good linear correlation between computed values of log β and the experimentally determined formation constants. Studies were performed on geometric isomers and hydration states of Zr-DFO as well as on 23 different complexes of a total of 17 different chelators including many examples described in this review (Figure 8).

**Figure 9:**
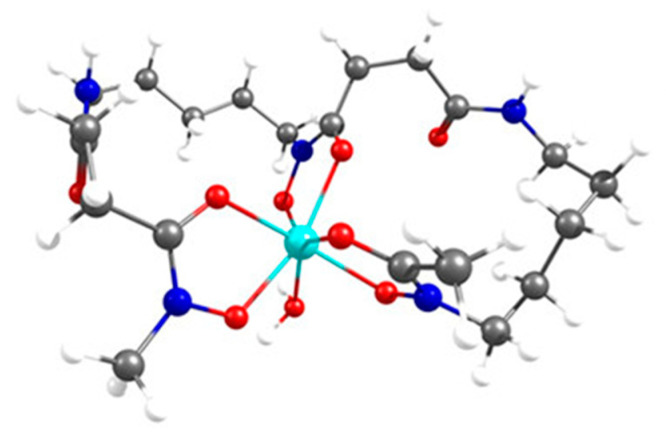
Structure of the DFT optimized geometric isomer of the seven-coordinate [Zr(DFO)(H_2_O)]^+^ complex in N-cis-cis orientation. Reprinted (adapted) with permission from [28]. Copyright {2020} American Chemical Society.

For Zr-DFO, a wide range of energetically similar geometric isomers with coordination numbers ranging from six to eight donor atoms in the first coordination sphere were predicted. Perhaps surprisingly, the calculation revealed the most stable Zr complex in solution is the seven-coordinate [Zr(DFO(H_2_O)]^+^ species in which the hydroxamate ligands adopts a *N*-*cis*-*cis* orientation (Figure 9). That linear hydroxamate chelators form different isomers was also observed by Patra et al. for Zr-DFO*-CO_2_H using NMR spectroscopy. It was claimed that the envelopment of the metal center by the ligand is dynamic. The statement was further substantiated by unusual fronting of the γ-HPLC peak corresponding to the radiolabeled peptide conjugates of DFO and DFO*, which was ascribed to the presence of different isomers of the radiometal complex [37].

Through the analysis of the different complexes the following trends could be identified by Holland [28]. First, as expected, octa-coordinated Zr complexes show higher log β values than hexa-coordinated ones (e.g., DFO*, oxoDFO* vs. DFO, DFO-O3, respectively). Secondly, DFO performs well and remains a good chelator for zirconium, however, it is outrun by a number of the new analogs (e.g., THPN, CTH36, DFO*, DFO-HOPO, oxoDFO*; the same seems to hold truth for 4HMS, DFOcyclo*, DFO2, which are described in this review, however, not included in the work of Holland). Third, ether groups in the DFO backbone (e.g., DFO-O3, oxoDFO*) do not only improve water solubility but also ligand flexibility, which leads in the highest predicted complex stability for oxoDFO*. Figure 8 illustrates a comparison of the theoretical thermodynamic stability (log β values) of 20 different Zr-complexes.

## 7. Conclusions

Hydroxamate-based chelators represent a promising class of compounds for the stable coordination of the metallic PET radionuclide ^89^Zr. They are therefore suitable candidates for applications in nuclear medicine (e.g., immunoPET). The hexadentate DFO is still the most used chelator today. It is approved by the FDA and EMA and shows good properties for ^89^Zr-radiolabeling in immunoPET applications. However, the in vivo stability of the complex ^89^Zr-DFO over time is suspected not to be sufficient, which has led to considerable research efforts worldwide for the development of better chelators. In fact, a number of the new octadentate hydroxamate-based chelators described herein exhibit improved properties (e.g., radiolabeling kinetics and/or complex stability) in comparison to the current clinical standard DFO.

Since the first reports of novel octadentate ^89^Zr-chelators in 2014 (DFO* [37] and HOPO [61]), a large number of new chelators for ^89^Zr has been developed and reported [74]. In most cases, their performance is assessed in comparison to the current standard DFO. While this practice is well justified, a direct comparison among the new chelators for the identification of the most promising candidate(s) for clinical translation is, with few exceptions [28,44,45,48], still pending. Along these lines, a standardization of in vitro experiments studying the stability of ^89^Zr-complexes would be highly desirable to facilitate a direct comparison of reported data. For this, important experimental parameters such as temperature, pH, ionic strength and the structure (EDTA, DTPA, or DFO) as well as the concentration of challenging ligands should be uniformed. Furthermore, in vivo data of biodistribution and/or quantitative PET imaging experiments with ^89^Zr-labelled compounds acquired at late time points (>24 h) are dearly needed for a full evaluation of the potential of new chelators. Unfortunately, such data is currently only available for a handful of the new promising compounds. Another important factor to consider is the availability of the chelators. Today, only bifunctional derivatives of DFO and DFO* are commercially available, which facilitates their use. In all other cases, in particular for comparison studies, the compounds are only available from the respective research groups, many of which have already shown a much-appreciated commitment to do so.

We anticipate that a successful successor of the currently used but not optimal chelator DFO for ^89^Zr-applications in nuclear medicine will be identified and translated into the clinic in due time. Concluding from this review, the most promising candidates are synthetically readily available octadentate and/or macrocyclic hydroxamate chelators. Whatever compound finally wins the race, it will represent an important milestone in radiopharmaceutical and nuclear medicinal sciences to the benefit of patients.
